# Histiocyte-rich Discoid Lupus Erythematosus: A Peculiar Perifollicular Distribution Histologically Mimicking an Acneiform Disorder

**DOI:** 10.7759/cureus.3310

**Published:** 2018-09-15

**Authors:** Ryan M McKee, Amanda F Marsch, Brian R Hinds

**Affiliations:** 1 Department of Dermatology, University of California San Diego, San Diego, USA

**Keywords:** discoid lupus erythematosus, histiocyte-rich, acneiform disorder, perifollicular

## Abstract

The histologic profile of discoid lupus erythematosus typically involves a vacuolar interface reaction with an associated superficial and deep perivascular infiltrate composed of lymphoplasmacytes. We present a unique case of discoid lupus erythematosus in which cluster of differentiation 68 (CD68) immunochemistry identified widely dispersed histiocytes. Few reports of histiocyte-rich cutaneous lupus erythematosus exist in the literature, and these lymphohistiocytic infiltrates, when present on the H-zone of the face, could be misconstrued as acne/rosacea. Our case demonstrates that cutaneous lupus erythematosus can present with a predominantly histiocytic infiltrate, a pattern dermatopathologists should be aware of to avoid non-recognition or misdiagnosis.

## Introduction

Discoid lupus erythematosus (DLE) represents a common form of cutaneous lupus erythematosus (CLE) that often prompts dermatologic consultation. The prototypical histologic finding of DLE is a vacuolar interface reaction with an associated brisk superficial and deep perivascular/periadnexal infiltrate composed of lymphoplasmacytes. We report a unique histiocyte-rich presentation of DLE that triggered a search for similar cases in the literature (PubMed: keywords included histiocyte, macrophage, lupus, and skin). In turn, lymphohistiocytic infiltrates in CLE have rarely been reported in the literature [1], and when arising on the H-zone of the face, this represents a recognizable guise that could be misconstrued as acne/rosacea.

## Case presentation

A 53-year-old woman with history of rheumatoid arthritis, interstitial lung disease, and Raynaud’s phenomenon presented with a photodistributed eruption on the scalp, face, arms, and chest two months after initiation of oral macitentan for pulmonary arterial hypertension. On exam, the patient had bright pink to violaceous, atrophic plaques on the cheeks, forehead, and nose. There was extension of erythematous crusted papules onto the upper extremities and chest. Initial biopsies from her left cheek and left upper arm showed an atrophic vacuolar interface dermatitis suggestive of connective tissue disease, favoring CLE. Laboratory studies revealed positive antinuclear antibodies (ANA, 1:640) and positive ribonucleoprotein (RNP). The following labs were negative or within normal limits: anti-Smith antibodies, aldolase, creatinine kinase, anti-Scl-70 antibodies, and anti-ds-DNA antibodies. A serum lipid panel was within normal limits. Clinicopathologic correlation led to a diagnosis of CLE, with drug-induced disease suspected. Macitentan was subsequently discontinued, topical steroids were initiated along with recommendations for photoprotection, and the eruption gradually improved over several months. 

**Figure 1:**
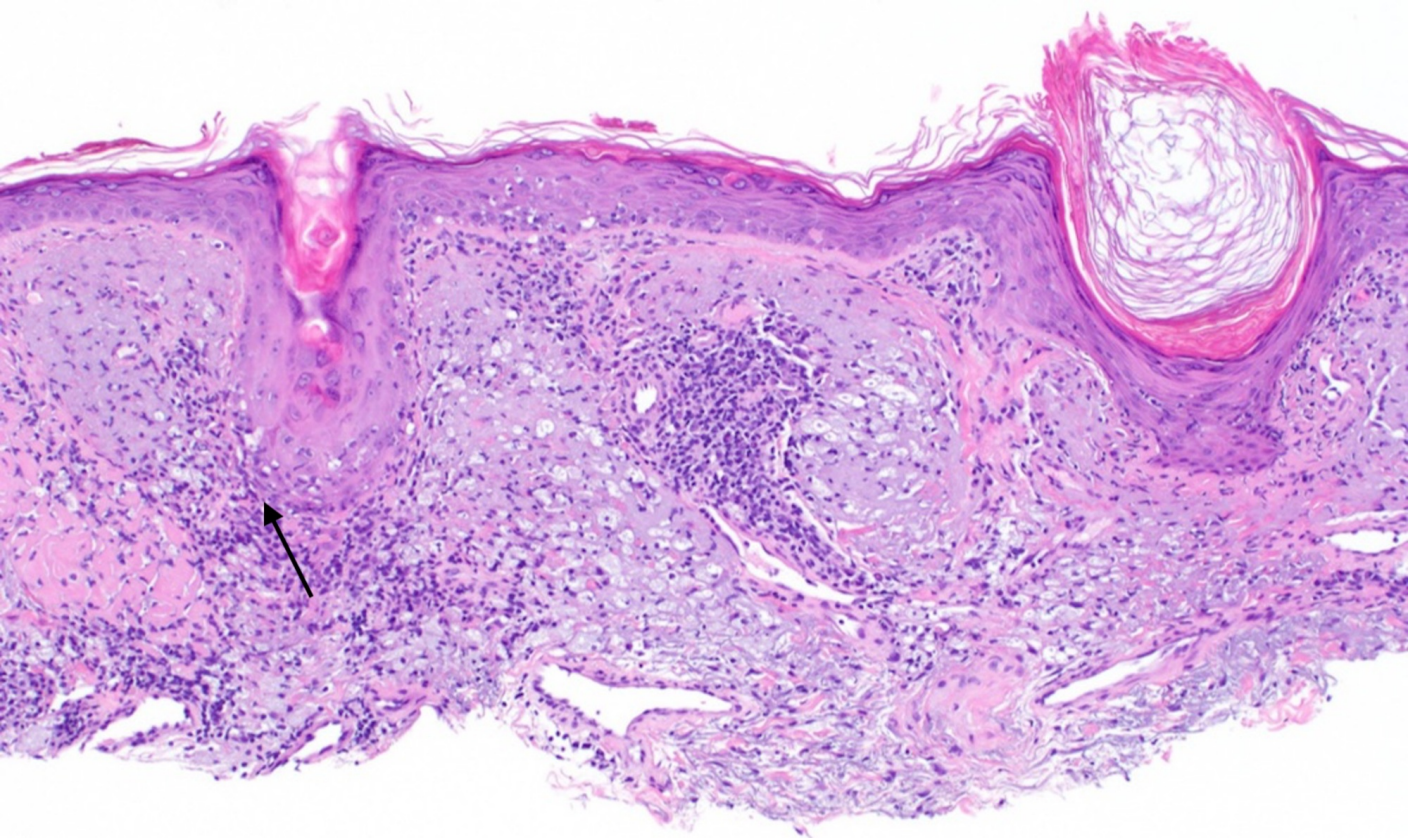
Perifollicular and perijunctional lymphocytic infiltrate with an associated vacuolar interface reaction. (H&E, 100X).

**Figure 2:**
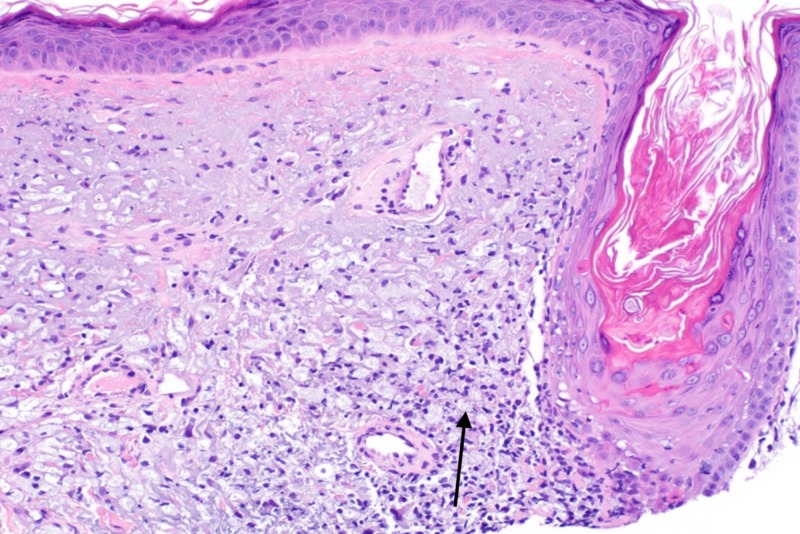
Loose dermal collections of pale cells with abundant, foamy cytoplasm are interspersed with the perifollicular lymphocytic infiltrate. (H&E, 200X).

**Figure 3:**
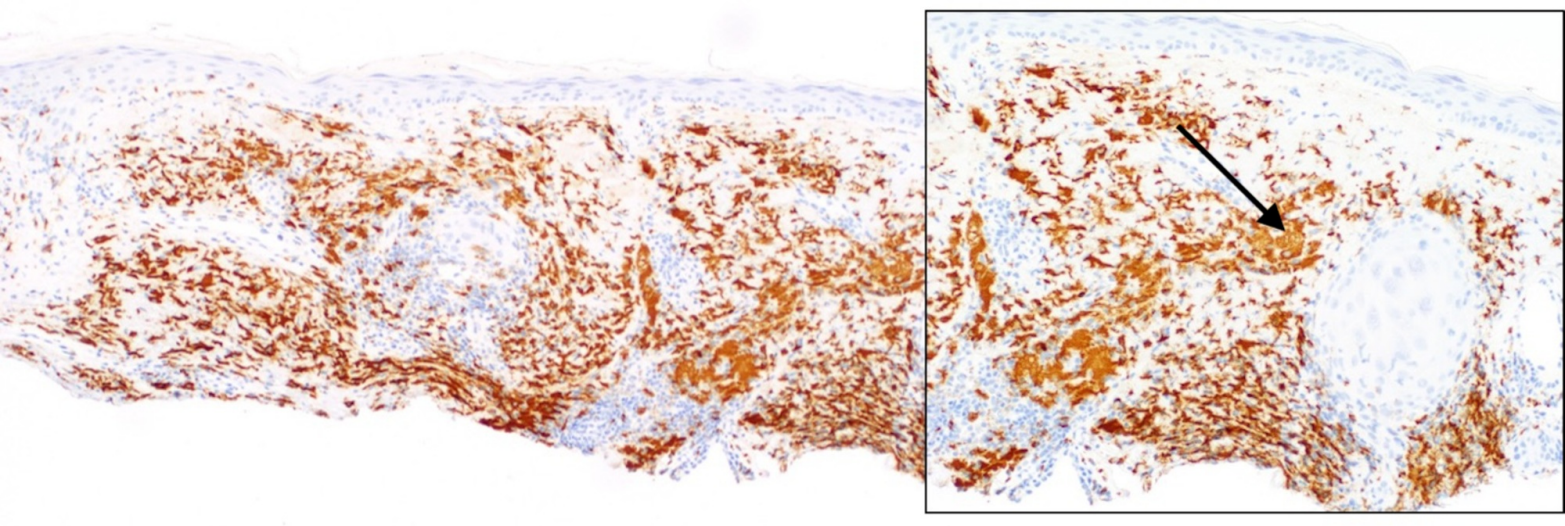
CD68 labels the pale vacuolated cells and also weakly labels some perijunctional and periadnexal lymphocytes (insert). (CD68, 100X & 200X).

**Figure 4:**
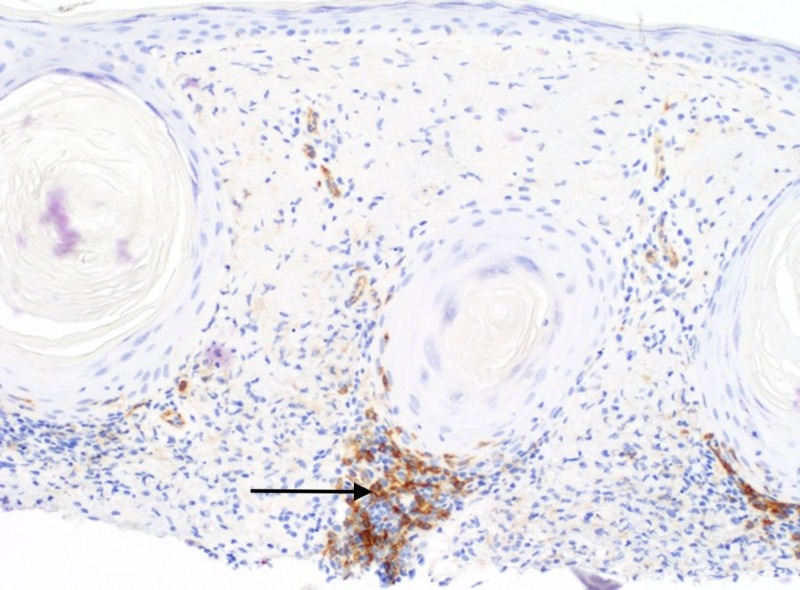
Small clusters and single cells corresponding to plasmacytoid dendritic cells in and around the follicular epithelium. (CD123, 200X).

**Figure 5:**
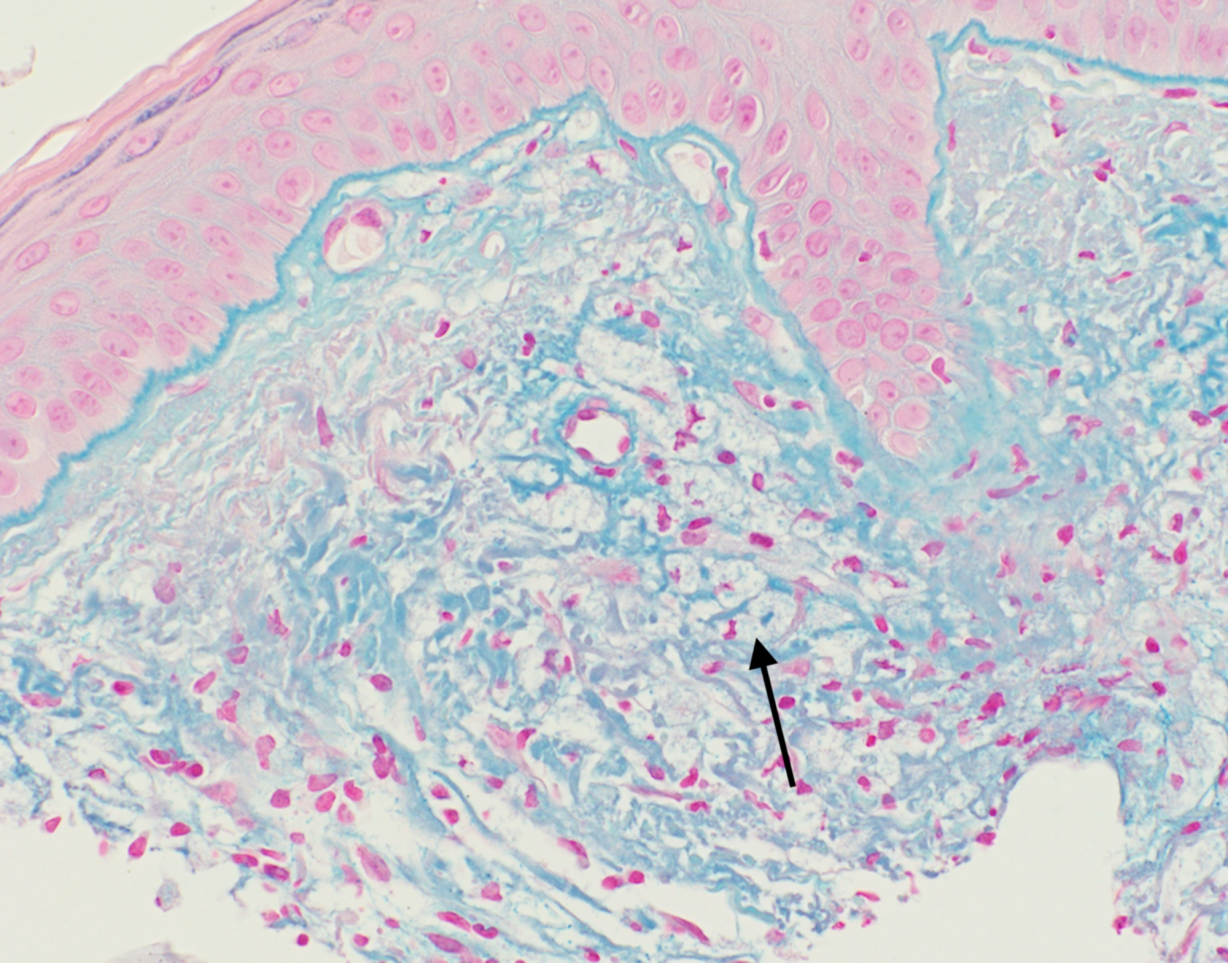
Colloidal iron stains showed interstitial dermal mucin, as well as labeling the intracytoplasmic material, substantiating histiocytes with engulfed ground substance. (Colloidal iron, 200X).

On follow-up exam, the pink plaque on the nasal dorsum persisted with telangiectasias, prompting a shave biopsy to exclude non-melanoma skin cancer. Histopathologic examination demonstrated patulous follicles intimately associated with a vacuolar interface reaction and a permeative perijunctional lymphocytic infiltrate (Figure 1). Immediately subjacent to this, enlarged pale cells with abundant, vacuolated cytoplasm were situated in loose perifollicular collections in an elastotic dermis (Figure 2). Limited immunohistochemistry, including S100, adipophilin, and cluster of differentiation (CD) 68 was performed to query the possibility of neurocristic/lipomatous, sebaceous, and histiocytic differentiation, respectively. S100 and adipophilin were negative in the cells of interest. CD68 labeled all of the pale vacuolated cells, confirming the presence of widely dispersed histiocytes (Figure 3). Of note, CD68 also co-labeled many of the lymphocytes (Figure 3). CD123 highlighted small clusters of perijunctional plasmacytoid dendritic cells (PDCs), but was negative in the histiocytic infiltrate (Figure 4). Colloidal iron showed interstitial dermal mucin and peppered the intracytoplasmic material, substantiating histiocytes with engulfed ground substance (Figure 5). A diagnosis of discoid lupus erythematosus was rendered. Of note, the patient has been seen in follow-up and the lesion resolved with an intralesional corticosteroid injection.

## Discussion

CLE is associated with a panoply of clinical and histopathologic findings with different characteristics ascribed to subtypes of acute (systemic lupus erythematosus), subacute (neonatal and drug-induced), and chronic (discoid, tumid, chilblain, panniculitis) disease. Rarely, interstitial histiocytic infiltrates may be observed in CLE producing microscopic mimicry reminiscent of granuloma annulare, and the infiltrate may also reportedly be perijunctional in distribution with round cells that engulf nuclear debris [2]. Although authorities have attributed such patterns to PDCs, CD123 immunohistochemistry has yet to be reported for these unusual circumstances. In the literature, prominent histiocytic infiltration has been observed in a neonate with known subacute cutaneous lupus erythematosus [1]. Histopathologic examination from an annular truncal plaque showed vacuolar interface reaction, papillary dermal edema, and leukocytoclasia, coupled with an interstitial and periadnexal mononuclear cell infiltration mimicking interstitial granulomatous dermatitis. The majority of the infiltrate expressed KP-1 (an anti-CD68 monoclonal antibody) and CD163, but S100 and CD1a were negative, typifying M2 macrophages. The cytomorphology of histiocytes was medium-sized mononuclear cells, as opposed to enlarged cells with abundant foamy cytoplasm. CD123 immunohistochemistry was not performed.

CD123 is a useful marker of plasmacytoid dendritic cells that shows increased labeling in CLE with dermal clusters or single cells distributed along the dermal epidermal junction [3]. For our case, CD123 was negative in the pale vacuolated cells, which excludes the possibility of PDC lineage, discounting the theory that histiocyte-like presentations of CLE are actually PDCs. Although negative in the pale vacuolated cells, CD123 did highlight small clusters of PDCs, which may aide in the recognition of cutaneous lupus and would avert a misdiagnosis of acneiform disorders, namely rosacea or acne. The histiocytic infiltration in this case adds to the peculiarity of this rare phenomenon, which is probably infrequently encountered. Of note, CD68 co-labeled the histiocytes and lymphocytes, which may be a pitfall in characterizing the immunophenotype of the infiltrate. This observation typifies previously reported heterogeneity of the antibody in non-myeloid cell types, perhaps reflective of an affinity for lysosomal protein unrestricted to macrophages [4].

## Conclusions

Although this case of lupus was felt to be drug-induced or exacerbated disease secondary to macitentan, it is unlikely for the histiocyte-rich infiltrate to be drug-related given that prior biopsies lacked histiocytes. It remains unclear whether this is a site-related phenomenon, provided that two of three cases to date were located on the head/neck (which is also the most common clinical location of DLE). The patient had no clinical or laboratory findings suggestive of xanthelesma or dyslipidemia, respectively. The patient had no prior history of biopsy or injections in the area so the possibility of artefact was sufficiently excluded both clinically and microscopically. In closing, to the uninitiated, lymphohistiocytic perifollicular infiltrates in seborrheic distribution could easily be misconstrued as acne/rosacea in a superficial tissue portion. Our case confirms that CLE can show a predominately histiocytic infiltrate and dermatopathologists should be aware of this pattern to avoid non-recognition or erroneous attribution to an acneiform disorder.
